# Accuracy of two orthodontic mini-implant templates in the infrazygomatic crest zone: a prospective cohort study

**DOI:** 10.1186/s12903-022-02285-0

**Published:** 2022-06-24

**Authors:** Li Su, Hui Song, Xiaofeng Huang

**Affiliations:** 1 Department of Stomatology, Beijing Xuanwu TCM Hospital, No. 13 Wan’ming Road, Xicheng District, Beijing, China; 2 Department of Stomatology, Beijing Friendship Hospital, Capital Medical University, No. 95 Yong’an Road, Xicheng District, Beijing, China

**Keywords:** Infrazygomatic crest, Mini-implant, Template

## Abstract

**Background:**

In the clinic, most computer-aided design and manufacturing orthodontic mini-implant guides are suitable for the position between the tooth roots, and few templates are designed and used for the infrazygomatic crest zone. In this study, we took into account the structure of the infrazygomatic crest and 3D printing technology, developed two kinds of templates, and evaluated their clinical effects.

**Methods:**

Seventeen patients who accepted 30 mini-implant insertions in the infrazygomatic crest were selected. According to different implantation methods, three groups were divided. In Groups A and B, the mini-implants were positioned with an A-type or B-type template designed by EXOCAD software. In Group C, the mini-implants were inserted by an experienced orthodontist without any guides. We simulate the bucco-palatal, mesio-distal, and vertical head positions in the Segma implant guide software and measure the deviation from the virtual design position of the mini-implant. The linear deviation of the mini-implant tip and cap and the angular deviation of the long axis of the mini-implant in the bucco-palatal direction, mesio-distal direction, and vertical direction were also measured. The results were statistically analysed by SPSS software.

**Results:**

The deviations of Group A and Group C’s miniscrew cap in the bucco-palatal direction, Group A and Group B, Group A and Group C’s miniscrew tip in the mesio-distal direction, and Group B and Group C’s miniscrew tip and cap in the vertical direction were statistically significant (*P* < 0.05). There was a significant difference in the deviations of Group A and Group C’s miniscrew tip and cap in the vertical direction (*P* < 0.01).

**Conclusions:**

In the vertical direction, the accuracy of implantation with the template is higher than that of the traditional method without the template to avoid piercing the maxillary sinus mucosa in the infrazygomatic crest zone.

## Background

Mini-implant implantation has become an essential method of controlling anchorage in the clinic and plays an important role in solving some difficult cases [1–3]. As a temporary bone anchorage, the mini-implant is easy to insert and remove. However, due to the limited space, there is a risk of injury to the roots [4, 5]. Therefore, the infrazygomatic crest zone is selected as an alternative implantation site in the clinic.

The infrazygomatic crest has a double-layered cortex and is close to the maxillary centre of the resistance, which is suitable for implantation and provides strong anchorage. However, it is adjacent to the maxillary sinus and tooth roots; therefore, we have to consider many factors, such as bone mass, the thickness of the buccal cortex and the relationship with the maxillary sinus and roots [6], before implantation in the infrazygomatic crest. However, previous research by our research group found that it is safe to penetrate the maxillary sinus within 1 mm [7].

To achieve precise implantation, implant templates are often used in the clinic. To date, most mini-implant templates have been designed for the position between the tooth roots [8–11] and palate [12, 13]. There are few studies on guides for the zygomatic alveolar ridge.

Computer-aided design and computer-aided manufacturing (CAD/CAM) technology has been widely used in the production of surgical implant guides [14, 15]. Using the same design idea as the implant CAD/CAM guide, we tried to design and manufacture a high-precision mini-implant template for the zygomatic alveolar ridge, especially in the vertical direction, to avoid injury to the maxillary sinus. In this study, cone beam computed tomography (CBCT) technology and laser scanning were integrated to establish a three-dimensional digital model that can display the jawbone, gingiva, mucosa and teeth to improve the accuracy of the template [8]. Based on these findings, we have developed two orthodontic mini-implant templates and assessed their accuracy.

## Methods

### Subjects

**Fig. 1:**
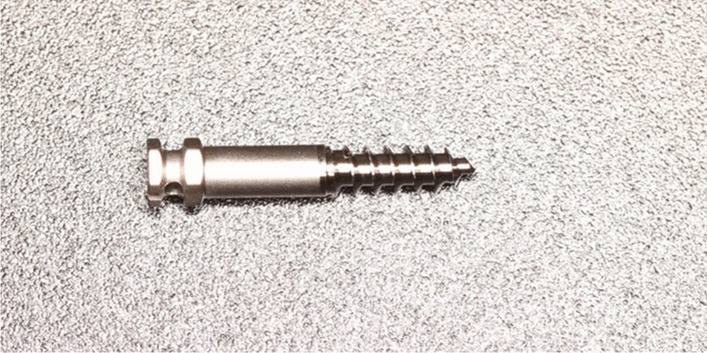
Mini-implant (Cibei, Ningbo, China, 2 mm in diameter and 13 mm in length)

This study was registered and approved by the biomedical ethics committee (ID: 2018-P2-054-01) of Beijing Friendship Hospital, Beijing, China. According to the sample size calculation formula [16], 17 patients who were about to accept 30 mini-implant insertions (Cibei, Ningbo, China, 2 mm in diameter and 13 mm in length) (Fig. 1) in the infrazygomatic crest were recruited. Subjects selected for this study had to fulfil the following inclusion criteria: (1) adult patients with fully developed roots and jaws; (2) orthodontic clinical design is the implantation of mini-implant in the infrazygomatic crest zone; (3) patients’ condition allows CBCT; and (4) no history of orthodontic treatment, periodontal disease, trauma or metal allergy.

Plaster models of each patient were obtained, and sufficient depth in the vestibular groove must be ensured when taking the impression with alginate.

All images were acquired with a CBCT machine (5G, version FP; NewTom, Verona, Italy) by experienced radiologists using standardized procedures. Cone-beam computed tomography (CBCT) can provide accurate 3-dimensional and high-resolution images of hard and soft tissues in the infrazygomatic crest and maxillary sinus, with a relatively low radiation dose and low cost [17, 18]. The data were reconstructed with cross-sectional slices at an interval of 0.3 mm. Clear CBCT.

views were obtained by adjusting the luminance and grey scale.

Three groups in all subjects were divided into Groups A, B, and C. In Groups A and B, the mini-implants were positioned with the help of an A-type or B-type template. In Group C, the same mini-implants were inserted by experienced orthodontists without any guides as the control group.

### Experimental design

**Fig. 2:**
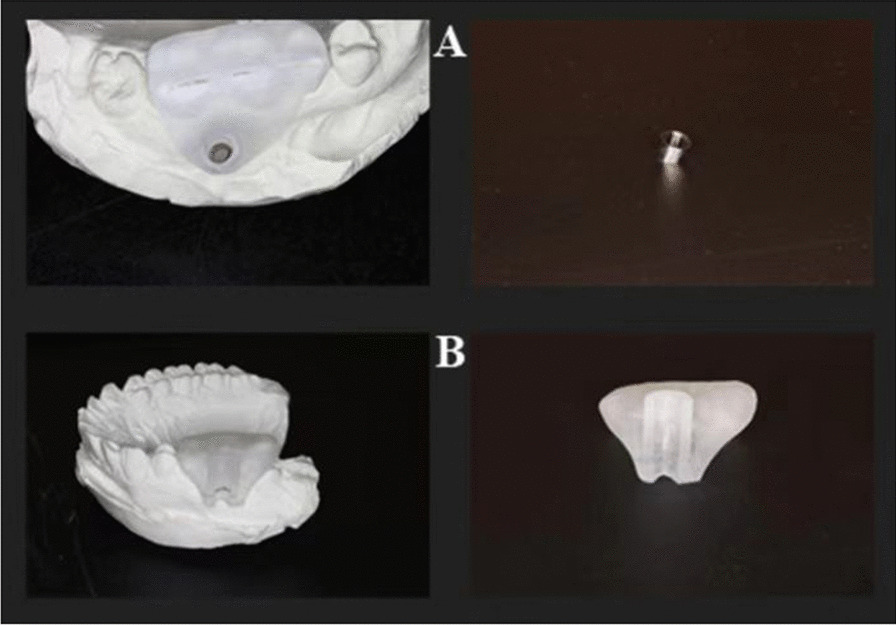
A-type (**A**) and B-type (**B**) templates

In the A-type template, a limit ring was designed to control the implantation depth. The template covers both the tooth and gingiva. Cracks were designed in the occlusal region, which made it convenient to remove the template after the mini-implant was implanted. The guide part of the B-type template adopts a semicircular structure so that the implantation angle can be changed after the mini-implant passes through a layer of bone cortex. A line marker indicated the implantation depth (Fig. 2).

The digital plaster models were obtained by scanning with a D700 scanner (3shape, Denmark). Both digital models and CBCT scan data were imported into Segma implant guide software (Beijing, China). The combination method is as follows: first, open the file in a way that only uses CBCT for implant design and adjust the density of anatomical landmarks such as the maxillary sinus, condylar foramen, or protrusions to an explicit level. Then, the teeth or jaw landmark points of the model and CBCT were selected to reconstruct a 3D model. After reconstructing the digital models of the bone teeth, we assessed the bone mass at the alveolar ridge and the distance from the tooth roots to the place of the mini-implants in the ideal virtual position, which has enough bone and avoids roots. According to the ideal virtual position of mini-implants, two kinds of implanting guides (A- and B-type templates) are designed by EXOCAD and printed by The EnvisionTEC Vida 3D printer (Germany) with photosensitive liquid resin material. After printing, the templates were rinsed with alcohol and subjected to secondary curing.

### Surgical procedure

**Fig. 3:**
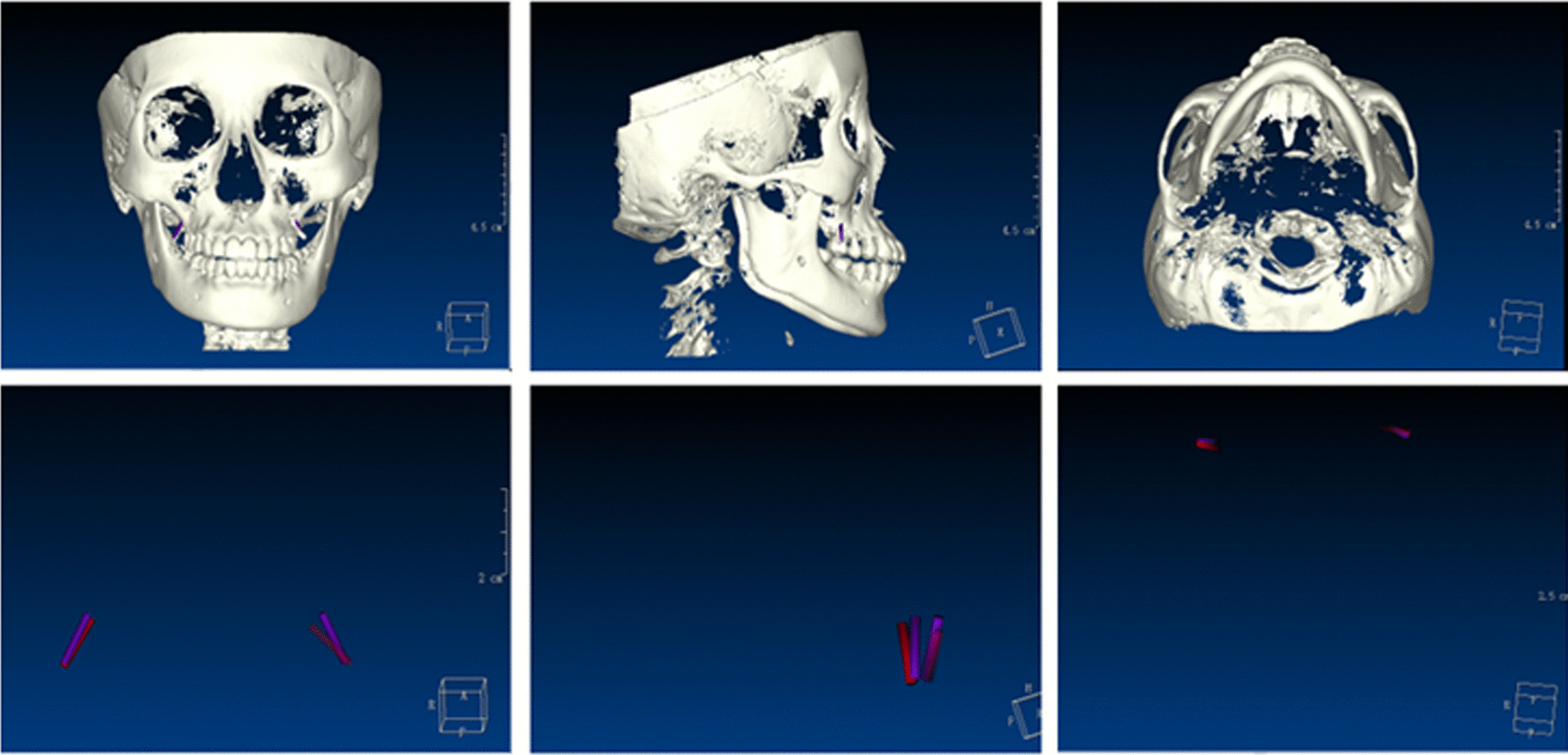
The 3D superposition digital model (the right side of the patient used the B-type guide plate to assist the implantation of the mini-implant, and the left side without any guides was used as the control group)

Before surgery, the patients were informed of the risks of surgery and signed an informed consent form. We checked the fit and stability of the mini-implant template before surgery. After local anaesthesia was given, the mini-implant was inserted under the guidance of the two kinds of templates by one orthodontist with 20 years of clinical experience in the implantation procedure. The same orthodontist operated the implantation without a template in the control group. The patient was scanned with CBCT again. The CBCT data were collected before and after the implantation of the mini-implant.The bucco-palata,mesio-distal and vertical positions in the Segma implant guide software were simulated (Fig. 3), and the deviation between the actual and virtual design positions of the mini-implant was measured.

### Statistical analysis

Two doctors with more than 2 years of clinical CBCT image analysis experience performed image superimposition and measurement: everyone measured images once, with a 1-day interval, for a total of 3 measurements, and the average value was taken.

The linear deviation of the mini-implant tip and cap and the angular deviation of the long axis of the mini-implant in the bucco-palatal, mesio-distal and vertical directions were measured. The results were analysed by the SPSS statistical package (version 19.0; IBM, Armonk, NY). The Kruskal–Wallis H test of the multiple independent samples method was used to compare the three groups of mini-implant᾽ deviation in the three directions.

For the values that did not conform to a normal distribution, the Bonferroni method was used to correct the significance level for pairwise comparisons. One-way ANOVA was used for values that conformed to a normal distribution.

## Results

The deviations of the miniscrew tip in the bucco-palatal direction in the three groups were 1.4383 ± 0.79025 mm (Group A), 3.2733 ± 1.56759 mm (Group B) and 3.0560 ± 2.75783 mm (Group C). The deviations of the miniscrew cap in the bucco-palatal direction in the three groups were 0.9383 ± 1.14341 mm (Group A), 2.5500 ± 1.50330 mm (Group B) and 3.8600 ± 5.27122 mm (Group C). The angular deviations of the long axis of the miniscrew at bucco-palatal were 4.5667 ± 2.36107° (Group A), 7.4889 ± 4.68467° (Group B) and 9.3667 ± 12.54920° (Group C). The deviations of the miniscrew tip in the mesio-distal direction in the three groups were 1.3700 ± 1.01453 mm (Group A), 3.3244 ± 1.45396 mm (Group B) and 2.9733 ± 1.63841 mm (Group C). The deviations of three group’s miniscrew cap in the mesio-distal direction were 1.6083 ± 1.91491 mm (Group A), 2.3789 ± 1.65812 mm(Group B) and 2.7687 ± 2.09217 mm(Group C).The angular deviations of the long axis of the miniscrew at mesio-distal direction were 7.7500 ± 8.14267° (Group A), 6.7111 ± 3.84820°,10.7533 ± 6.49724°(Group C).The deviations of three groups’ miniscrew tip at vertical direction were 0.5450 ± 0.60879 mm (Group A), 0.9722 ± 0.81269 mm(Group B) and 3.2853 ± 2.43316 mm (Group C).The deviations of three group’s miniscrews cap at vertical direction were 0.6150 ± 0.67299 mm (Group A), 1.1078 ± 0.81558 mm(Group B) and 3.9620 ± 5.81445 mm (Group C), respectively. The angular deviations of the long axis of the mini-implant in the vertical direction were 9.5167 ± 10.42332° (Group A), 10.6444 ± 9.11785° (Group B) and 14.6867 ± 8.21461° (Group C) (Table 1).

**Table 1:** Descriptive statistics of| the deviations of three groups’ miniscrew

Variable	Group A (n = 6)			Group B (n = 9)			Group C (n = 15)			H-value	*P*-value
	Mean ± SD	95% confidence interval		Mean ± SD	95% confidence interval		Mean ± SD	95% confidence interval			
		Minimum	Maximum		Minimum	Maximum		Minimum	Maximum		
Miniscrew tip at BP (mm)	1.4383 ± 0.79025	0.6090	2.2677	3.2733 ± 1.56759	2.0684	4.4783	3.0560 ± 2.75783	1.5288	4.5832	5.593	0.061
Miniscrew cap at BP (mm)	0.9383 ± 1.14341	−0.2616	2.1383	2.5500 ± 1.50330	1.3945	3.7055	3.8600 ± 5.27122	0.9409	6.7791	7.057	0.029*
Miniscrew angle at BP (°)	4.5667 ± 2.36107	2.0889	7.0445	7.4889 ± 4.68467	3.8879	11.0898	9.3667 ± 12.54920	2.4172	16.3162	1.075	0.584
Miniscrew tip at MD (mm)	1.3700 ± 1.01453	0.3053	2.4347	3.3244 ± 1.45396	2.2068	4.4421	2.9733 ± 1.63841	2.0660	3.8807	6.380	0.041*
Miniscrew cap at MD (mm)	1.6083 ± 1.91491	−0.4012	3.6179	2.3789 ± 1.65812	1.1043	3.6534	2.7687 ± 2.09217	1.6101	3.9273	2.424	0.298
Miniscrew angle at MD (°)	7.7500 ± 8.14267	−0.7952	16.2952	6.7111 ± 3.84820	3.7531	9.6691	10.7533 ± 6.49724	7.1553	14.3514	3.941	0.139
Miniscrew tip at VD (mm)	0.5450 ± 0.60879	−0.0939	1.1839	0.9722 ± 0.81269	0.3475	1.5969	3.2853 ± 2.43316	1.9379	4.6328	14.176	0.001**
Miniscrew cap at VD (mm)	0.6150 ± 0.67299	−0.0913	1.3213	1.1078 ± 0.81558	0.4809	1.7347	3.9620 ± 5.81445	0.7421	7.1819	14.346	0.001**
Miniscrew angle at VD (°)	9.5167 ± 10.42332	−1.4219	20.4553	10.6444 ± 9.11785	3.6358	17.6530	14.6867 ± 8.21461	10.1376	19.2358	3.137	0.208

*BP* bucco-palatal direction, *MD* mesio-distal direction, *VD* vertical direction

*P < 0.05; **P < 0.01

There was a significant difference in the deviations of the miniscrew cap in the bucco-palatal direction and the miniscrew tip in the mesio-distal direction (*P* < 0.05). The deviations of the miniscrew tip and cap in the vertical direction were significantly different (*P* < 0.01) (Table 1).

The deviations of Group A and Group C’s miniscrew cap in the bucco-palatal direction, Group A and Group B, Group A and Group C’s miniscrew tip in the mesio-distal direction, and Group B and Group C’s miniscrew tip and cap in the vertical direction were statistically significant (*P* < 0.05). There was a significant difference in the deviations of Group A and Group C’s miniscrew tip and cap in the vertical direction (*P* < 0.01) (Tables 2, 3**).**

**Table 2:** Bonferroni test result of the deviation of the miniscrew tip at mesio-distal direction

Variable	GroupA–Group B		GroupA–Group C		GroupB–GroupC	
	Mean difference	*P*-Value	Mean difference	*P*-Value	Mean difference	*P*-Value
Miniscrew tip at MD	−1.95444	0.019*	−1.60333	0.034*	0.35111	0.580

*MD* mesio-distal direction

*P < 0.05; **P < 0.01

**Table 3:** One way ANOVA test result of the deviation of the miniscrew

Variable	F-value			*P*-value		
	Group A–Group B	Group A–Group C	Group B–Group C	Group A–Group B	Group A–Group C	Group B–GroupC
Miniscrew cap at BP	− 2.168	− 2.580	− 0.246	0.090	0.030*	1.000
Miniscrew tip at VD	− 0.815	− 3.312	− 2.776	1.000	0.003**	0.017*
Miniscrew cap at VD	− 0.731	− 3.288	− 2.853	1.000	0.003**	0.013*

*BP* bucco-palatal direction, *MD* mesio-distal direction, *VD* vertical direction

*P < 0.05; **P < 0.01

## Discussion

Since the end of the twentieth century, the clinical application of mini-implant anchorage has become a research hotspot in the field of orthodontics. At present, the CAD/CAM guide for implants provides a new idea for the design of high-precision mini-implant templates:3D reconstruction and registration technology assist in the design of implantation positions, using the inverse technique to design the digital file of the template and the template made by rapid prototyping technology (RP).

To design a high-precision mini-implant template, an accurate digital model should be first established. There are direct and indirect ways of building a digital model. The direct method is the use of a small penetrating optical scanning probe to obtain the surface morphology of soft and hard tissues such as teeth and gingiva in the patient's mouth, omitting the operations of making impressions and plaster models. The indirect method needs to make a plaster model first and then apply related technologies to reconstruct the digital model after obtaining a three-dimensional model image by the scanning protocol.

The most commonly used three-dimensional modelling methods include optical scanning technology, CT scanning technology, and intraoral direct scanning technology. For dental crowns, optical scanning is more accurate. CBCT has obvious advantages in tooth root and jaw scanning reconstruction [8]. Therefore, we used the method of optical scanning combined with CBCT.

3D printing technology reconstructs a three-dimensional design model through computer aided design (CAD) software or reverse engineering. The 3D printer processes the layered materials according to the data read by the software to form a three-dimensional model. Currently, commonly used rapid prototyping technologies in the dental field include stereolithography appearance (SLA), fused deposition modelling (FDM), selective laser sintering (SLS), stereo inkjet printing (inkjet-based system, IBS), and low-temperature deposition modelling (LDM). This experiment used digital light processing (DLP) technology. The software Segma implant guide uses sequential point registration and global registration to establish a high-precision three-dimensional integrated model. The deviation analysis function of the software is used to detect the 3D deviation between the two models automatically. The chromatogram shows that the registration accuracy of the three-dimensional cone beam CT model and the three-dimensional optical scanning model is high, and the registration accuracy can reach 0.1 mm. The measurement accuracy can reach 0.001 mm.

Regarding the accuracy of the mini-implant template, Bae [11] reported that the angular deviation of the mini-implant implanted by the guide was a median of 3.14°, and the mesio-distal deviations in the coronal and apical areas were medians of 0.29 mm and 0.21 mm, respectively. Liu [19] reported that the position deviations in the mesio-distal, vertical, and buccopalatal directions were 0.42, 0.47, and 0.59 mm at the tip, respectively. The mini-implant deviations of the angles from the planned position in the distomesial, vertical, and buccopalatal directions were 1.2°, 1.3°, and 1.6°, respectively. The two mini-implant templates in this study are more accurate than the templates previously reported by Liu [19] in the vertical direction.

The application of templates can increase the accuracy of mini-implant insertion, thereby reducing damage to adjacent normal structures. The interval alveolar bone between teeth roots, especially between the maxillary first molar and the second premolar, is a frequently used mini-implant site in orthodontic clinics. However, the width of alveolar bone between tooth roots varies greatly, and it easily hurts the roots. Therefore, accurate implantation is necessary. Previous studies have also suggested the use of mini-implant guides when implanting between roots [8–13] to avoid damaging the roots in the mesial and distal direction.

In this study, two implant guides were designed to control the implant depth, which can avoid the influence of the zygomatic alveolar ridge when implant anchorage was implanted to a certain extent. We hope that the mini-implant can ensure sufficient bone retention without hurting the maxillary sinus.

The infrazygomatic crest has sufficient bone mass and a double layer of cortical bone, but it is adjacent to the maxillary sinus. Therefore, when implanting mini-implants in this area, the depth of insertion should be considered, avoiding injury to the maxillary sinus. Our previous research found that it is safe for mini-implants to penetrate the maxillary sinus within 1 mm [7]. The result provided guidance on the vertical limitation of mini-implants in the position of the infrazygomatic crest zone.

In this study, two implant templates were designed to control the implantation depth, which can avoid the influence of the maxillary sinus. We hope that the mini-implant template can ensure sufficient bone retention without hurting the maxillary sinus to ensure the safety of adjacent tissues and the stability of the mini-implant.

## Conclusions

By using CAD/CAM technology, we designed and manufactured two kinds of high-precision mini-implant templates for the infrazygomatic crest zone, specifically to control the vertical insertion depth to avoid injury to the maxillary sinus. The new designs can increase the tissue safety, which might provide a better guarantee for the application of implant anchorage in the infrazygomatic crest.

## Data Availability

All data generated or analysed during this study are included in this published article.
